# Negative phototaxis in the photosymbiotic sea anemone Aiptasia as a potential strategy to protect symbionts from photodamage

**DOI:** 10.1038/s41598-023-44583-9

**Published:** 2023-10-19

**Authors:** Mariko Kishimoto, Sebastian G. Gornik, Nicholas S. Foulkes, Annika Guse

**Affiliations:** 1 National Institute for Basic Biology, Nishigonaka 38, Myodaiji, Okazaki, Aichi 444-8585 Japan; 2 Centre for Organismal Studies, Heidelberg University, Im Neuenheimer Feld 230, 69120 Heidelberg, Germany; 3 Present Address: Department of Translational Genomics, Medical Faculty, University of Cologne, Weyertal 115b, 50931 Cologne, Germany; 4 Institute of Biological and Chemical Systems, Karlsruhe Institute of Technology, 76344 Eggenstein-Leopoldshafen, Germany; 5 Present Address: Faculty of Biology, Ludwig-Maximilians-Universität München, Großhadernerstr. 2, 82152 Planegg-Martinsried, Germany

**Keywords:** Ecology, Behavioural ecology, Ecophysiology, Microbial communities, Symbiosis

## Abstract

Photosymbiotic cnidarians generally seek bright environments so that their symbionts can be photosynthetically active. However, excess light may result in a breakdown of symbiosis due to the accumulation of photodamage in symbionts causing symbiont loss (bleaching). It is currently unknown if photosymbiotic cnidarians sense light only to regulate spawning time and to facilitate predation, or whether they also use their light-sensing capacities to protect their symbionts from photodamage. In this study, we examined how the sea anemone Aiptasia changes its behaviour when exposed to excess light. We reveal that Aiptasia polyps, when carrying symbionts, contract their bodies when exposed to high light intensities and subsequently migrate away in a direction perpendicular to the light source. Interestingly, this negative phototaxis was only evident under blue light and absent upon UV, green and red light exposure. Non-symbiotic Aiptasia did not exhibit this light response. Our study demonstrates that photosymbiotic Aiptasia polyps display negative phototactic behaviour in response to blue light, and that they also can perceive its direction, despite lacking specialized eye structures. We postulate that Aiptasia uses blue light, which penetrates seawater efficiently, as a general proxy for sunlight exposure to protect its symbionts from photodamage.

## Introduction

Most cnidarians need specific lighting conditions to thrive^1,2^. Corals and sea anemones, for example, which often form an endosymbiotic relationship with dinoflagellates of the family Symbiodiniaceae for nurture^3^, seek out shallow, bright waters; while non-symbiotic cnidarians prefer deeper, darker environments^4^. Similarly, distinctive light preferences are also found in photosymbiotic and non-symbiotic individuals within single cnidarian species^5^. For example, previous studies in the photosymbiotic sea anemone Aiptasia showed that anemones move towards white light^5,6^ and low intensity blue light^5^. Thus, photosymbiotic cnidarians may have evolved strategies to continuously seek out ideal light environments to optimize the photosynthesis rates of their symbionts^6^. Failure to find optimal light environments can be disastrous: stressful light conditions (i.e. short wavelength, high-intensity light) damage the photosystems of symbionts^7^ resulting in a breakdown of symbiosis^8,9^. However, studies assessing behaviour in response to light are sparse. To better understand how photosymbiotic cnidarians evolved to protect their symbionts from light stress, here we studied Aiptasia and investigated whether it displays differential phototactic behaviour depending on symbiosis status and the nature of the light it encounters.

## Results and discussion

**Figure 1:**
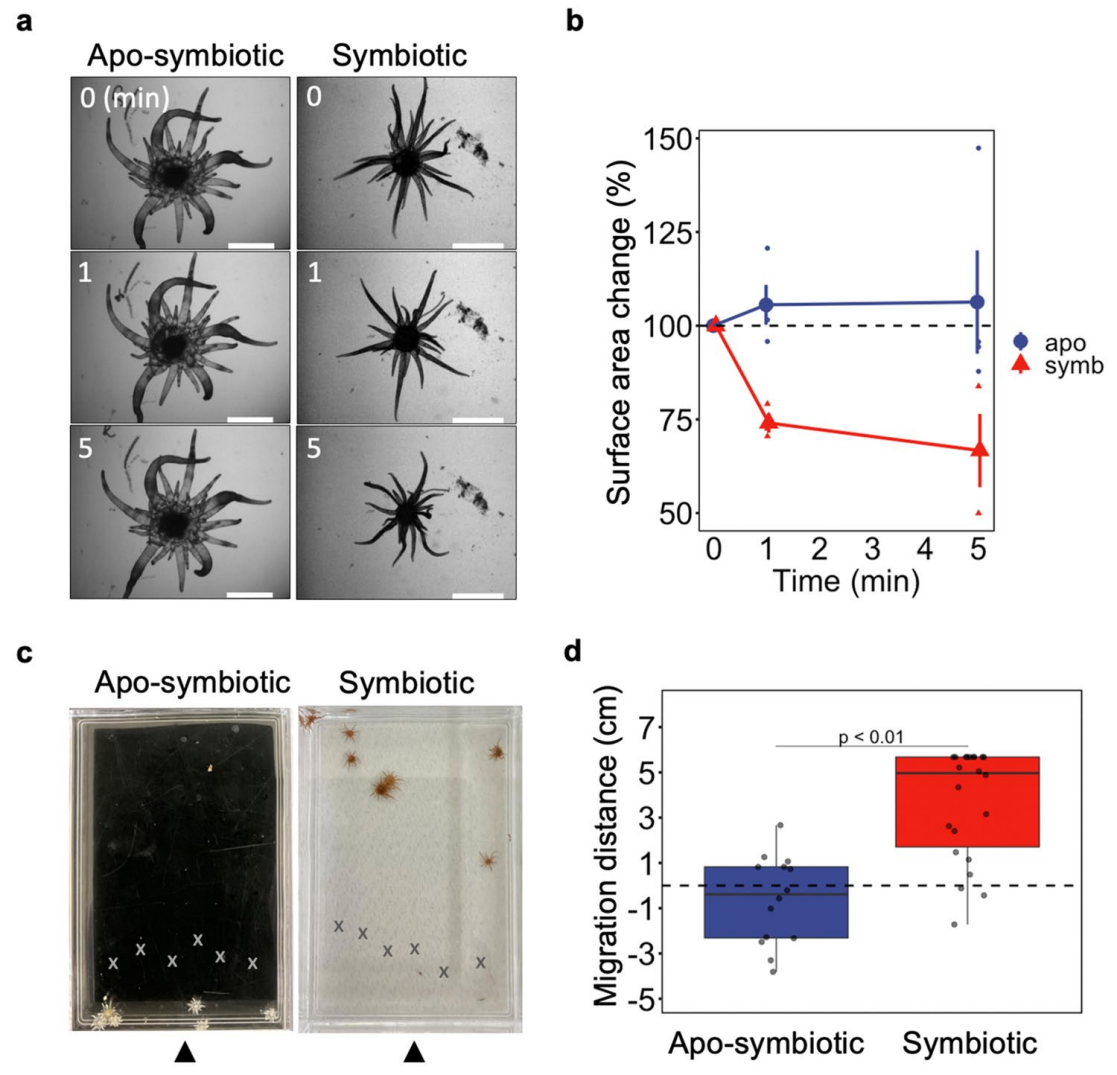
Effect of light on symbiotic and non-symbiotic Aiptasia polyps. (**a**) Time-lapse observations of Aiptasia polyps after 0, 1 and 5 min of treatment under the irradiation of multi-coloured LED light (400, 430, 450, 470, 500 and 680 nm; at a PFD of 200 µmol photons m^–2^ s^–1^) which is four times higher than their normal culture conditions and the wavelength mimics the natural light environment of the ocean at a depth of 10 m. Scale bar = 10 mm. (**b**) Changes in the 2-dimensional surface area from the images of Aiptasia monitored during irradiation with the same multi-coloured LED light. Values are shown as individual points and also as mean ± SE from 3 or 4 independent experiments. (**c**) Images of apo-symbiotic and symbiotic Aiptasia following exposure to multi-coloured LED light for 24 h (at a PFD of 70 µmol photons m^–2^ s^–1^). The starting positions of polyps are indicated with cross labels (X). The arrows indicate the direction of the light source. (**d**) The migration distance was measured following LED irradiation for 24 h. Presented data are from three independent experiments and each point represents an individual. The box and line represent the quartiles and median, respectively. Positive values indicate migration away from the light source. The p-value to compare between symbiotic and apo-symbiotic individuals was calculated by Welch’s t-test.

We first exposed non-symbiotic and photosymbiotic Aiptasia polyps to polychromatic light (comprising 400, 430, 450, 470, 500 and 680 nm) that mimics the natural light environment of the ocean at a depth of 10 m, at a PFD (Photon Flux Density) of 200 µmol photons m^–2^ s^–1^, which is four times higher than their normal culture conditions. Photosymbiotic Aiptasia contracted their bodies within the first minute of exposure (Fig. 1a) resulting in a decrease in their surface area (Fig. 1b). Non-symbiotic Aiptasia failed to respond even when exposed for up to five minutes (Fig. 1a). We then tested phototactic behaviour over longer time scales (24 h) using the same set-up. Here, photosymbiotic polyps migrated away from the light source (Fig. 1c,d) while non-symbiotic polyps remained immobile (Fig. 1c,d). The mean migration distance was significantly larger in photosymbiotic polyps (p-value = 0.0000025).

**Figure 2:**
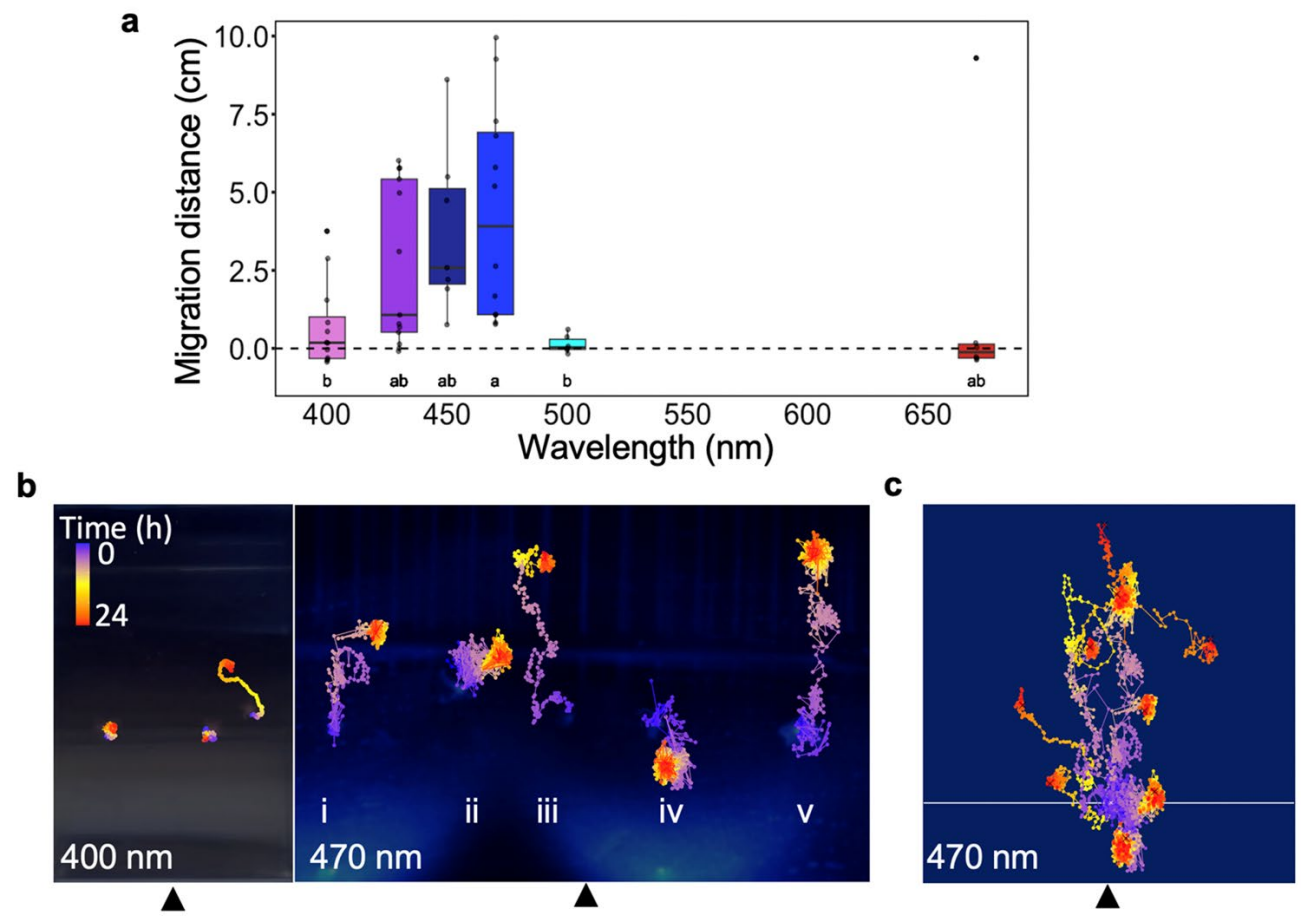
Wavelength specificity of the negative phototaxis in photosymbiotic Aiptasia. (**a**) Migration distance was measured following LED irradiation for 24 h at different wavelengths (400, 430, 450, 470, 500 and 680 nm). Presented data are from three independent experiments and each point represents an individual. The box and line represent the quartiles and median, respectively. Positive values indicate migration away from the light source. The results of one-way ANOVA (F = 3.753, p = 0.00581) and Tukey’s post-hoc test are shown under the boxplots. (**b**) Trajectory traces of symbiotic Aiptasia polyps during irradiation using monochromatic LED lights (as indicated; using a PFD of 70 µmol photons m^–2^ s^–1^). The position of each polyp was monitored every 5 min using a time-lapse camera. Each observation is represented by a single dot, and all observations were merged into a single photograph. The arrows indicate the direction of the light source. (**c**) Merged migration traces of nine Aiptasia individuals monitored at 470 nm. The horizontal line indicates the starting position of each polyp. The arrow indicates the direction of the light source. The final positions of the polyps are indicated with black cross labels (X).

We next tested whether this phototactic behaviour is induced by specific monochromatic light. At a PFD of 70 µmol photons m^–2^ s^–1^, we observed substantial migration at 430, 450 and 470 nm only. Migration was subdued at 400 nm, and absent at 500 and 680 nm (Fig. 2a). Wavelengths between 500 and 680 nm (green and orange light) were not tested. Finally, to understand the temporal nature of this blue light-induced migration we exposed photosymbiotic polyps to either 470 nm (blue light; migration peak) or 400 nm (violet light; no/little migration) light while using time-lapse photography. The migration distance was significantly affected by wavelength (one-way ANOVA (F = 3.753, p = 0.00581)). Polyps exposed to 470 nm for 24 h moved constantly and travelled a considerable distance (up to 5 cm; Fig. 2b,c); while at 400 nm polyps only moved intermittently over short distances. Generally, the trajectories recorded at 470 nm were perpendicular to the light source (Fig. 2b–i,iii,v,c). However, occasionally polyps moved in circles or toward the light (Fig. 2b–ii,iv), which may indicate initial phototactic searching behaviours. It seems, however, that these individuals never progressed beyond this initial behaviour. Maybe for these individuals the light cues we provided were not easily detected and thus they failed to respond. Nevertheless, it is evident that a phototactic avoidance behaviour of photosymbiotic Aiptasia polyps is induced by high-intensity blue light.

In cnidarians, blue light affects numerous behaviours including spawning^10–12^, locomotion ^13,14^, swimming patterns of larvae^15^, and tentacle contraction^16^. Here, our study shows that hosts display negative phototactic behaviour away from blue light sources and that they can also perceive its direction, despite lacking specialized eye structures. Since the sensitivity to blue light is so widespread amongst Cnidaria, they likely share a common blue light sensing system that was already present in their last common ancestor. Indeed, multiple photoreceptor protein families (notably opsins and cryptochromes) are shared amongst all cnidaria^17^, but their importance in blue light detection and their role in photosymbiosis is largely unknown. Interestingly, in Aiptasia*,* three opsins are exclusively expressed at high levels in symbiotic polyps^17^ and we hypothesize that the observed negative phototaxis of photosymbiotic Aiptasia away from blue light is conveyed by at least one of these opsins. It seems that these opsins enable Aiptasia polyps to sense and ultimately avoid light environments with a high blue light component. However, without testing this hypothesis through in vitro characterization, knock-out studies or comparable ablated expression of these opsins, this remains purely speculative. Alternatively, since aposymbiotic animals are kept in constant darkness, their photoreceptors may not be expressed and therefore these animals lack a response to light. However, Gornik et al.^17^ showed that all Aiptasia photoreceptors have a baseline expression and are actively expressed even in constant darkness. Thus, a complete lack of photoreception in aposymbiotic animals is unlikely.

Another possible mechanism that could explain the negative phototaxis observed in photosymbiotic Aiptasia may sense light-induced damage in symbionts and, via a cascade of signals, stimulates polyp migration. It is conceivable that a host cell could perceive reactive oxygen species (ROS) that leak out of photodamaged symbionts following light stress^8,9^. The host cell could then communicate this ROS signal to the surrounding tissue eventually causing the polyp to move away from the damaging light source. While such a sensory system may exist, we nevertheless postulate that it does not play a role in the negative phototaxis of symbiotic Aiptasia based on the wavelength and timing of this behaviour. We show that symbiotic Aiptasia react only to blue light and within the first minute of exposure, while UV makes the highest impact on the symbiont photosystem and even in highly photosensitive strains such as *Breviolum psygmophilum* (CCMP2459, formerly IST-type B2), it takes at least 30 min of intense UV irradiation to obliterate photosynthetic activity^7^.

It was recently described that photosymbiotic Aiptasia perceives both high and low-intensity white light to orchestrate optimal conditions for the photosynthesis of their symbionts by moving toward the light source^6^. Our results now show that Aiptasia also exhibits negative phototaxis away from blue light sources. Maintenance of optimal light conditions is crucial for stabilizing symbiosis between cnidarians and their symbionts^3,18,19^. Excessive light can strain the photosystem of symbionts and cause photodamage^7,8^. We thus postulate that Aiptasia polyps possess negative phototaxis to protect their symbionts from photodamage. It is of considerable advantage for Aiptasia to use blue light as a proxy for the sunlight, since, for example, red and UV light (UV-A and UV-B) are absorbed easily in seawater. In contrast, blue light (400–500 nm) penetrates ocean water more efficiently, traveling as deep as 500 m^20^. It seems that instead of evolving a more complex photoprotective system that would monitor different wavelengths of polychromatic light, Aiptasia has developed a sensory system for photoprotection that is tuned to the most prevalent light in the marine environment.

## Methods

### Cultures and growth conditions

The sea anemone *Exaiptasia pallida* (strain H2, commonly called Aiptasia) polyps were provided by Professor John Pringle (Stanford University). Symbiotic individuals in this study are clonal and were never bleached. Thus they retained their original *S. minutum* (SSB01) symbiont strain. The propagated clonal Aiptasia polyps were cultured in filtered artificial seawater (REISEA marine 2; IWAKI) at 25 °C under a cycle of 12 h of fluorescent lamps at a photon flux density (PFD) of 50 μmol photons m^–2^ s^–1^ and 12 h of darkness. Animals were fed freshly hatched *Artemia* nauplii larvae once a week. Symbiotic polyps were incubated at 34 °C in the dark for 1 month to obtain aposymbiotic individuals that eliminated all symbionts. The aposymbiotic animals were subsequently kept in constant darkness at 25 °C and fed freshly hatched *Artemia* nauplii larvae once a week. Before all light treatment tests, aposymbiotic animals were incubated under a cycle of 12 h of fluorescent lamps at a photon flux density (PFD) of 50 μmol photons m^–2^ s^–1^ and 12 h of darkness.

### Light treatment

Aposymbiotic and photosymbiotic Aiptasia polyps were placed in 6 well-plates with 5 ml of artificial seawater for treatment with a polychromatic LED lamp (BlueHarbor, SPECTRA/SP200) and exposed at a PFD of 200 µmol photons m^–2^ s^–1^), for 5 min under a fluorescence microscope (Leica M165FC) with 200 × magnification. The LED setting was set as Simple Setting; depth: 10 M; Power; 100% and consisted of 400 (range of 405–410 nm), 430 (range of 430–435 nm), 450 (range of 450–455 nm), 470 (range of 470–475 nm), 500 (range of 495–500 nm) and 680 nm (range of 660–680 nm) lamps which mimics the natural ocean light environment. The model number and the brand of each LED lamp are the following: 400 nm (LTPL-C034UVH405; LITE-ON), 430 nm (LTPL-C034UVH430; LITE-ON), 450 nm (XP-E2; CREE), 470 nm (XP-E2; CREE), 500 nm (EP-U4545K; Epileds) and 680 nm (Dual chips; Epileds). The detailed information about the lamps including spectrums is available on the brand websites and at the website of BlueHarbor (https://www.blueharbor.co.jp/en).

Images of polyps were taken at the start of the experiment and at 1 min and 5 min during treatment under the microscope. The surface areas of the tentacles and the oral rings were manually selected using the polygon tool in ImageJ and the sizes of the areas were measured in ImageJ. Finally, the relative difference between sample measurements from the 2 treatment groups (apo- vs photosymbiotic) was calculated. We performed 3 (symbiotic), and 4 (apo-symbiotic) independent experiments using 3 individuals per test. For long exposures, Aiptasia polyps were held in water tanks (10 × 5 × 2 cm) filled with 50 ml of artificial seawater (REISEA marine 2; IWAKI). The tank was then placed in an incubator at 25 °C with the polychromatic LED lamp positioned horizontally on the wall of the incubator. Prior to starting each exposure, the light intensity was measured, and tanks were positioned so that the polyps were exposed to a PFD of 70 μmol photons m^–2^ s^–1^ light. The position of each polyp was measured again after 24 h of incubation. We performed three independent experiments and we used 4–6 individuals for each test, as above. When testing long exposures of monochromatic light, we used the same LED lamp but only the lamps of a single specific wavelength were switched on. Tanks were positioned as the polyps were exposed to a PFD of 70 μmol photons m^–2^ s^–1^ light. Other settings were the same as described above for the long-term exposure test. We performed three independent experiments and used 2–3 individuals for each test. When comparing two samples we used Welch’s *t* test to account for unequal variances and unequal sample sizes. For comparison of multiple samples we used one-way ANOVA.

### Trajectory analysis of Aiptasia migration

Individual frames recorded using 5 min time-lapse (described above) were converted to monochrome TIFF files using FIJI. Following this, in each frame of a series, the position of each individual polyp was detected as higher intensity spots and the coordinates of these spots were recorded to a CSV file. By tracing the closest spots among the time series, all spots were grouped into trajectories of each individual polyp. The time series of coordinates for each individual polyp was then plotted as a point and line graph using ggplot2 package in R 4.2.0. An image of a standard water tank was placed as background to aid visualisation.

## Data Availability

All data generated or analyzed during this study are included in this published article.
